# Assessment of paraclinical characteristics in peri- and postmenopausal bleeding women: is there a correlation between hemoglobin levels and ultrasonic indices?

**DOI:** 10.1016/j.jtumed.2022.10.011

**Published:** 2022-11-15

**Authors:** Phuc N. Nguyen, Van T. Nguyen

**Affiliations:** a Department of High-risk Pregnancy, Tu Du Hospital, Ho Chi Minh City, Viet Nam; b Tu Du Clinical Research Unit (TD-CRU), Tu Du Hospital, Ho Chi Minh City, Viet Nam; c Department of Obstetrics and Gynecology, Hue Medical College, Hue University, Thua Thien Hue, Viet Nam

**Keywords:** نزيف الرحم غير الطبيعي, سرطان بطانة الرحم, انقطاع الطمث, بعد انقطاع الطمث, أمراض الرحم داخل تجويف الرحم, Abnormal uterine bleeding, Endometrial cancer, Perimenopause, Postmenopause, Uterine intracavitary pathologies

## Abstract

**Objectives:**

Vaginal bleeding is a common symptom of uterine intracavitary pathologies in perimenopausal and postmenopausal women, which leads to anemia. However, findings regarding the relationship between hemoglobin level and sonographic parameters remain limited. The aim of this study was (1) to investigate the histopathological findings of uterine intracavitary pathologies, hemoglobin concentrations and basic sonographic parameters, and (2) to evaluate their correlation among Vietnamese women with perimenopausal and postmenopausal bleeding.

**Methods:**

This was a prospective study at Hue University Hospital and Hue Central Hospital from June 2016 to June 2019. The study enrolled 150 women older than 40 years with abnormal uterine bleeding. All patients underwent blood count testing and transvaginal ultrasound.

**Results:**

Moderate to severe anemia was observed at a higher frequency in women with perimenopausal bleeding (58.1%) than postmenopausal bleeding (10.0%). The most common abnormality resulting in severe anemia was endometrial hyperplasia (70.8%), which was followed by endometrial cancer (4.2%). The uterine size, intrauterine mass, and endometrial thickness differed substantially between the benign and malignant groups. The study found significantly a weak negative correlation between hemoglobin concentration (g/L) and uterinelength, the anteroposterior diameter of uterine corpus in the overall study (r = −0.37, r = −0.32, respectively, P < 0.05); a moderate negativecorrelation between hemoglobin concentration and the largest diameter of intracavitary mass‑shaped lesion in the perimenopausal group (r = −0.4, P < 0.05).

**Conclusion:**

Overall, histopathological results, hemoglobin concentration and basic sonographic parameters should be combined in evaluating intrauterine abnormalities in women with perimenopausal and postmenopausal bleeding. Ultrasonic indices of uterine size may be used to determine the prognosis of anemia in uterine intracavitary pathologies. However, further studies are needed to confirm these findings.

## الملخص

**أهداف البحث:**

نزيف الرحم غير الطبيعي هو عرض شائع لأمراض الرحم داخل تجويف الرحم لدى النساء في فترة ما قبل انقطاع الطمث وبعد انقطاع الطمث مما يؤدي إلى فقر الدم. ومع ذلك ، فإن النتائج حول العلاقة بين مستوى الهيموجلوبين ومعلمات تخطيط الصدى لا تزال محدودة. كان الهدف من هذه الدراسة هو (1) دراسة النتائج المرضية لأمراض الرحم داخل تجويف الرحم ، وتركيز الهيموجلوبين بالإضافة إلى بعض المعايير الأساسية للموجات فوق الصوتية. و (2) لتقييم ارتباطهم بين النساء الفيتناميات مع نزيف ما قبل انقطاع الطمث وبعد انقطاع الطمث.

**طرق البحث:**

كانت هذه دراسة مقطعية في مستشفى اتش يو ومستشفى اتش سي من يونيو 2016 إلى يونيو 2019. شملت الدراسة 150 امرأة فوق 40 عاما مصابات بنزيف الرحم غير الطبيعي. تم فحص جميع المرضى عن طريق تعداد الدم وخضعوا للموجات فوق الصوتية عبر المهبل.

**النتائج:**

تعرضت النساء المصابات بنزيف ما قبل انقطاع الطمث لفقر دم معتدل إلى شديد (58.1٪) أكبر من النساء المصابات بنزيف ما بعد انقطاع الطمث (10.0٪). كان الشذوذ الأكثر شيوعًا الذي أدى إلى فقر الدم الوخيم هو تضخم بطانة الرحم (70.8٪) ، يليه سرطان بطانة الرحم (4.2٪). اختلف حجم الرحم والكتلة داخل الرحم وسماكة بطانة الرحم اختلافًا كبيرًا بين المجموعات الحميدة والخبيثة. وجدت الدراسة ارتباطًا سلبيًا ضعيفًا متوسطًا بين تركيز الهيموجلوبين (جم / لتر) وطول الرحم ، القطر الأمامي الخلفي لجسم الرحم في الدراسة الشاملة ؛ علاقة سلبية معتدلة بين تركيز الهيموجلوبين وأكبر قطر من آفة الرحم داخل التجويف في مجموعة ما قبل انقطاع الطمث.

**الاستنتاجات:**

بشكل عام ، يجب الجمع بين النتائج النسيجية المرضية وتركيز الهيموغلوبين والمعايير الأساسية للتصوير بالموجات فوق الصوتية لتقييم التشوهات داخل الرحم في النساء اللائي يعانين من انقطاع الطمث وبعد انقطاع الطمث. يمكن استخدام مؤشرات الموجات فوق الصوتية لحجم الرحم للتنبؤ بفقر الدم في أمراض الرحم داخل تجويف الرحم. هناك حاجة إلى مزيد من الدراسات لتأكيد هذه النتائج.

## Introduction

The menstrual cycle is the result of the balance among proliferation, decidualization, hemorrhage and regeneration. An imbalance in any one of these stages can lead to abnormal uterine bleeding (AUB).^1^ AUB is defined as abnormal menstrual flow in terms of volume, duration, regularity or frequency. AUB occurs in one-third of women during the reproductive period and accounts for more than 70% of all gynecologic consultations in perimenopausal and postmenopausal women.^2^, ^3^, ^4^ Among AUBs, uterine intracavitary bleeding commonly has structural causes including endometrial polyps (EP), endometrial hyperplasia (EH), subendometrial fibroids (EF) and endometrial cancer (EC).^5^

Beyond evaluation of clinical features, subclinical investigations including histopathological endpoints are crucial in assessing perimenopausal and postmenopausal bleeding (PMB).^6^^,^^7^ The level of blood loss may be neglected or estimated inaccurately in clinical examination.^8^ Moreover, a complete blood count aids in determining the exact level of anemia. Beyond laboratory assessments, sonography is also an appropriate modality to initially investigate the structure of the uterus.^9^^,^^10^ Ultrasonography has been described as giving gynecologists a second set of eyes, because it can reveal changes in uterine size as well as structural abnormalities in the uterine cavity.^11^, ^12^, ^13^ These non-invasive findings are more acceptable first-line imaging modalities than invasive modalities such as endometrial biopsy or hysteroscopy.^4^^,^^14^ Moreover, some studies have attempted to find correlations between the uterine size or endometrial thickness and the hemoglobin (Hb) concentration; however, the results remains limited in the literature.^15^^,^^16^ If these findings are significant, clinicians can assess the prognosis of early anemia and provide earlier interventions, such as administration of iron for preventing the suspected anemia afterwards. Thus, uterine intracavitary pathologies that result in abnormal uterine bleeding should be evaluated carefully with the available tools in perimenopausal and postmenopausal women, particularly in middle-low income countries such as Vietnam.

## Materials and Methods

### Study design and population

This prospective interventional study was conducted according to the ethics committee of our university and was approved by our institutional review board (IRB approval 1435/QĐ-ĐHYD). All patients provided written informed consent to participate. This prospective interventional study was conducted at Hue University Hospital and Hue Central Hospital between June 2016 and June 2019.

### Inclusion criteria

All women older than 40 years with complaints of abnormal uterine bleeding were included. The patients underwent blood count testing and evaluation of uterine sonographic parameters including uterine length (mm), anterior-posterior (AP) diameter of the uterine corpus (mm), largest diameter of the uterine intracavitary mass lesion (mm) and endometrial thickness (mm). Subsequently, these results were compared with the histopathological results.

### Exclusion criteria

**Figure 1:**
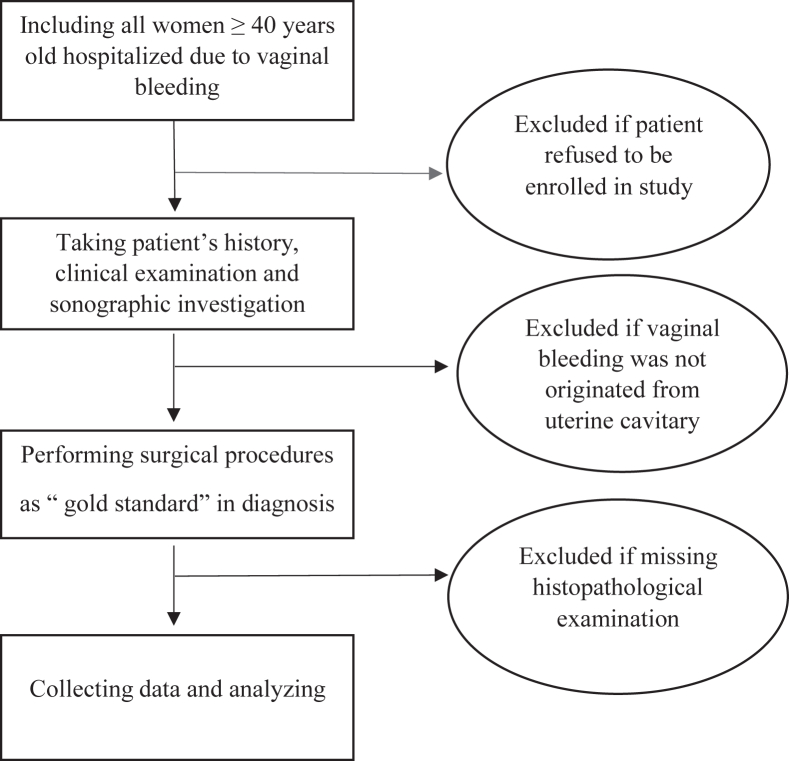
Study flowchart.

Patients with chronic anemia due to thalassemia or hematologic malignancy, and those who had received previous blood transfusions before blood count testing were excluded. Patients with bleeding associated with pregnancy, hormone replacement therapy, tamoxifen, coagulation disorders, bleeding not originating from the uterine cavity, abnormalities associated with the cervix, iatrogenic bleeding and lack of information on the study protocol were also excluded (Figure 1).

Overall, a total of 150 women, including 90 perimenopausal women and 60 postmenopausal women, were recruited and participated in this study.

All patients underwent thorough recording of disease history, general examination, gynecological examination, cervical cytology (Pap smear) and laboratory investigations (complete blood count (Sysmex-XP-300 and Sysmex-XN series XN-1000 Automated Hematology Analyzer, Japan), liver function tests, kidney function tests and coagulation profile). Then abdominal and transvaginal sonography was performed for all patients with a 5–7.5 MHz endovaginal transducer and abdominal probe (Samsung WS80A, HS70A, Korea). One of four sonographers with more than 5 years of experience was randomly allocated to each participant. During the exploration, the sonographer first determined the image of the uterus, occupying 2/3 of the screen, to access the clearest image. One of four sonographers was automatically assigned to perform the ultrasonic scan.

### Measurement of basic sonographic parameters (Figure 2)

#### Uterine size (mm)

The longitudinal dimension (uterine length) in sagittal sections (the long axis) was measured from the highest fundal point in the midline to the corresponding midline cervical point (external os).

**Figure 2:**
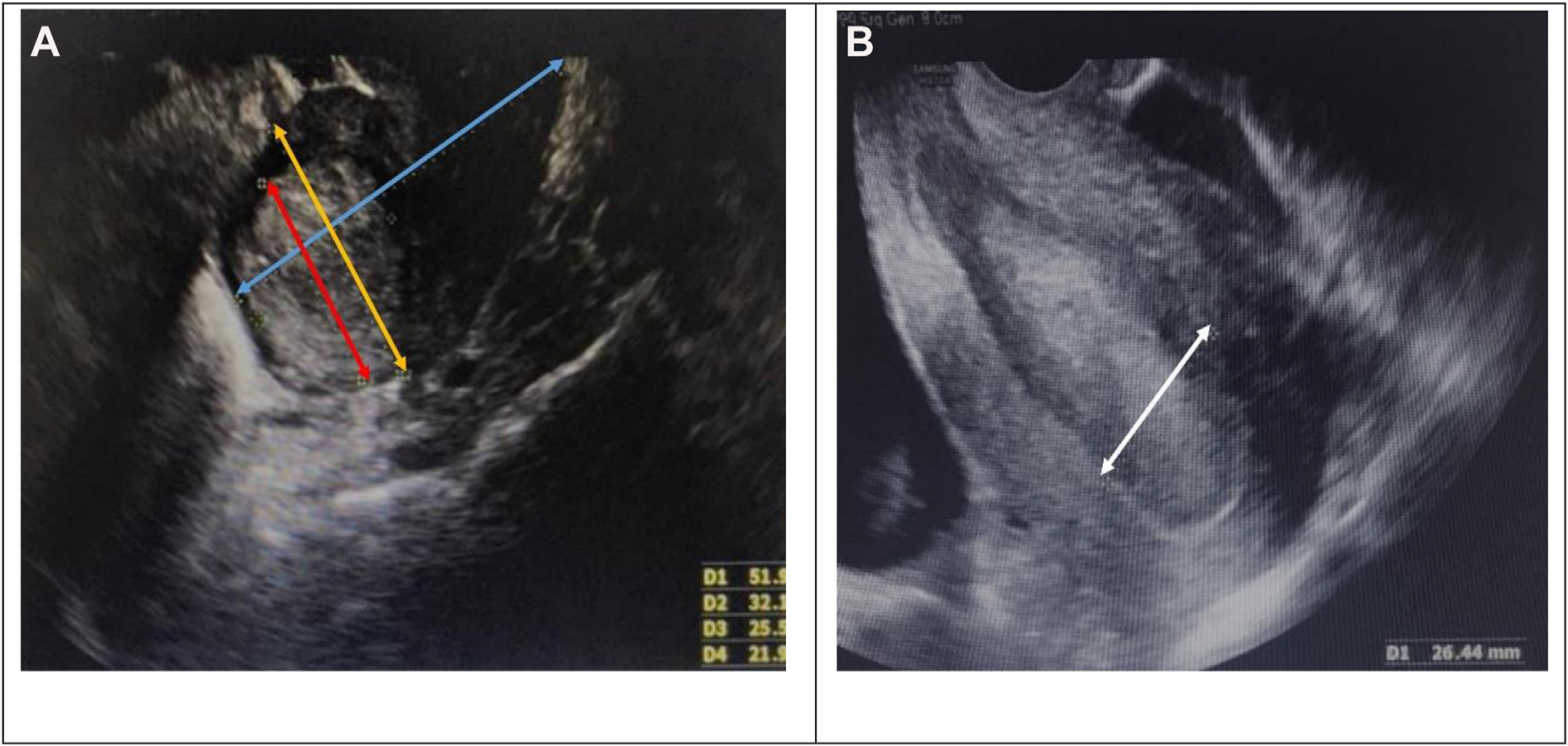
Basic measurements of uterine parameters on sonography. A: Uterine length (blue line), uterine width (yellow line), largest diameter of uterine intracavitary mass (red line). B. Endometrial thickness (white line).

The AP diameter, in sagittal sections at 90° to the longitudinal plane at the widest fundal dimension was measured. This depth was measured from the anterior to the posterior wall, perpendicular to the length in the midsagittal plane.

The width was measured in the transverse (semi-coronal) plane from one corne to the opposite corne (maximum width).

#### Endometrial thickness (mm)

The endometrium thickness depends on the menstrual phase. ET was measured in the long axis or sagittal plane, with the entirety of the endometrial lining through to the endocervical canal in view. The measurement is among the thickest echogenic areas from one basal endometrial interface across the endometrial canal to the other basal surface. ET measurement did not include hypoechoic myometrium or intrauterine fluid. In the postmenstrual period, the endometrium is particularly thin, and the basal layer shows a single echogenic layer.

#### Intrauterine lesion mass (mm)

The greatest dimension of lesions was measured at the maximum diameter (mm).

Before abdominal ultrasound, patients had full bladders; regardless for transvaginal ultrasound, the bladder was required to be empty. The patients lay in supine position. All dimensions were measured at least twice to allow for observer error, and the mean was recorded. The dimensions were determined at their widest points and recorded in millimeters (mm) to the nearest decimal.

All patients underwent interventional procedures such as endometrial biopsy, hysteroscopy, myomectomy or hysterectomy, as decided on the basis of the medical protocol currently applied at our hospital.

Data were recorded from the patient files and classified according to the following:

- Continuous variables included uterine parameters (uterine length (mm), AP diameter of the uterine corpus (mm), largest diameter of the uterine intracavitary mass lesion (mm), endometrial thickness (mm)) and concentration of Hb (g/l). Anemia was assessed according to the classification of the World Health Organization in 2011 for non-pregnant women (WHO 2011).^17^
- Categorical variables included histopathological result types, uterine intracavitary pathologies and level of anemia.

Patients received blood transfusion if Hb ≤ 70.0 g/l or ≤ 80.0 g/l with symptoms of anemia.

The histopathological results were collected within 1–2 weeks after the intervention and were considered the “gold standard” for diagnosis of uterine intracavitary pathologies.

### Statistical analysis

Statistical analyses were performed in Statistical Package for the Social Sciences (SPSS) version 20.0 (IBM Corp., Armonk, NY, USA), and P < 0.05 was considered statistically significant.

Spearman and Pearson's correlation (depending on the distribution of data) were used to determine the correlation coefficient (r) between two variables, which ranged from −1 to +1, indicating a negative and positive correlation, respectively. An | r | value > 0.8 indicated a strong correlation, 0.4 ≤ | r | < 0.8 indicated a moderate correlation, and | r | < 0.4 indicated a weak correlation.

### List of abbreviations

AUB, abnormal uterine bleeding; EC, endometrial cancer; EP, endometrial polyp; EH, endometrial hyperplasia; EF, sub-endometrial fibroids; PMB, postmenopausal bleeding; UIPs, uterine intracavitary uterine pathologies.

## Results

In the group of perimenopausal women, almost all cases had benign pathology, including EH (66.7%), EP (15.6%) and EF (13.3%). EC was observed in only 2/90 cases (2.2%). Regarding postmenopausal women, EH had the highest rate (51.7%) and was followed by EC (38.3%), EP (3.3%) and EF (1.7%) (Table 1).

**Table 1:** Frequencies and percentages of uterine pathologies.

Pathology			Perimenopause		Postmenopause		Total	
			n	%	n	%	n	%
Intracavitary uterine	Benign	Polyp^a^	14	15.6	2	3.3	16	10.7
		Sub-endometrial fibroma	12	13.3	1	1.7	13	8.7
		EH^b^	60	66.7	30	51.7	91	60.7
		Other^c^	2	2.2	3	5.0	5	3.3
	Malignant	Endometrial cancer^d^	2	2.2	23	38.3	25	16.6
		**Total**	**90**	**100.0**	**60**	**100.0**	**150**	**100.0**
Extracavitary uterine		Cervical polyp	1	1.1%	1	1.7%	2	1.3%
		Intramural fibroid^e^	7	7.8%	3	5.0%	10	6.7%
		Adenomyosis	0	0.0%	1	1.7%	1	0.7%
		**Total**	**8**	**8.9%**	**5**	**8.3%**	**13**	**8.6%**

a Among EP, 13/16 cases (81.3%) were adenomatous polyps; 3/16 cases (18.7%) were fibroepithelial polyps.

b Among EH, 68/91 cases (74.7%) were hyperplasia without atypia; 23/91 cases (25.3%) were EH with atypia.

c Others included endometrite, endometrial hemorrhage and normal proliferative endometrium.

d Among EC, 22/25 (88.0%) cases were adenomatous EC; 3/25 cases (12.0%) were other types. Classification grades were grade 1: 3/25 (12%), grade 2: 3/25 (12.0%) and grade 2: 3/25 (12.0%); 16 cases were not identified.

e We found ten cases of intramural fibroids along with intra-cavitary uterine pathology, with sizes ranging from 10 to 39 mm; the fibroid number in one case varied from 1 to 3,and was classified by FIGO 3-6.^5^

The mean ± SD of Hb concentration in perimenopausal women was lower than that in postmenopausal women (99.41 ± 25.13 vs 123.55 ± 12.05 g/l, p < 0.001) (Table 2). The most common pathology causing moderate and severe anemia was EH, at 53.1% and 70.8%, respectively. Severe anemia was not markedly present in the EC group; the mean Hb concentration in the EC group was 122.28 g/l (Table 3). Uterine size on ultrasonography did not significantly differ between the benign and malignant groups in the overall study and in perimenopausal women (Table 4). In contrast, the uterine size was greater in the malignant group than the benign group in postmenopausal women. Intrauterine mass lesions were present in 47 cases. The largest masses were associated with submucosal fibroids, then, with pathologies of EC, EP and EH, respectively (Table 5).

**Table 2:** Levels of anemia in the perimenopausal and postmenopausal bleeding groups.

Level of anemia^a^	Bleeding group					
	Perimenopause		Postmenopause		Overall study	
	n	%	n	%	n	%
Severe (Hb < 80 g/l)	24	26.7	0	0.0	24	16.0
Moderate (Hb: 80–109 g/l)	27	30.0	6	10.0	33	22.0
Mild (Hb: 110–119 g/l)	19	21.1	10	16.7	29	19.3
Normal (Hb > 120 g/l)	20	22.2	44	73.3	64	42.7
**Total**	**90**	**100**	**60**	**100.0**	**150**	**100.0**

a Using Chi-squared test to determine if there is a diﬀerence between the levels of anemia and intracavitary uterine pathologies: Chi-squared test *χ*2 = 44.96, P < 0.0001.

**Table 3:** Moderate and severe anemia in patients with uterine intracavitary pathology.

Intra-cavitary pathology	Anemia				
	Severe Hb < 80 (g/l)		Moderate Hb: 80–109 (g/l)		Concentration of Hb (g/l)
	n	%	n	%	$\bar{X}$ ± SD (minimum–maximum)
EC	1	4.2	2	6.1	122.28 ± 17.14 (63–141)
EP	3	12.5	5	15.2	101.94 ± 26.15 (41–137)
EF	3	12.5	5	15.2	97.00 ± 22.66 (61–130)
EH	17	70.8	17	51.5	109.13 ± 24.48 (45–147)
Other^e^	0	0.0	4	12.1	96.00 ± 10.00 (87–110)
**Total**	**24**	**100.0**	**32**	**100.0**	109.07 ± 23.99 (41–147)

e Endometrial atrophy, endometritis, proliferative endometrium, etc.

EC: Endometrial cancer, EP: Endometrial polyp, EF: Endometrial fbroid,EH: Endometrial hyperplasia, SD: Standard deviation, Hb: Hemoglobin.

**Table 4:** Sonographic features in the benign and malignant groups.

Size (mm)	Perimenopause		Postmenopause		Total	
	Benign	Malignant	Benign	Malignant	Benign	Malignant
***Anterior-posterior diameter (corpus thickness)***	54.7 ± 9.0	49.5 ± 0.7	41.6 ± 10.5	49.7 ± 12.3	50.8 ± 11.2	49.7 ± 11.8
$\bar{X}$ ± SD min–max	54.5 ± 9.0 (34–79)		44.7 ± 11.8 (26–74)		50.6 ± 11.3 (26–79)	
p	0.426		0.008		0.665	
***Longitudinal diameter (uterine length)***	83.8 ± 13.9	70.0 ± 7.1	66.3 ± 13.8	82.4 ± 22.9	78.7 ± 16.0	81.4 ± 22.2
$\bar{X}$ ± SD minimum–maximum	83.5 ± 13.9 (50–129)		72.5 ± 19.4 (40–140)		79.1 ± 17.1 (40–140)	
p	0.166		0.005		0.566	
***Endometrial thickness***	14.20 ± 7.45	22.5 ± 14.85	9.43 ± 6.61	24.18 ± 10.60	12.83 ± 7.46	23.99 ± 10.58
$\bar{X}$ ± SD minimum–maximum	14.38 ± 0.81 (2–39)		14.02 ± 1.42 (2–46)		14.25 ± 0.73 (2–46)	
p	0.129		0.001		0.001	

P-values from independent samples t-tests.

**Table 5:** Sizes of uterine intracavitary mass lesions.

Intracavitary pathology	Size			
	$\bar{X}$ ± SD (mm)	Smallest (mm)	Largest (mm)	n
EC	44.23 ± 20.94	10	85	13
EP	22.18 ± 11.67	4	46	14
EF	45.38 ± 25.68	14	110	13
EH	13.17 ± 4.90	8	20	7

Interestingly, we found a negative correlation between uterine size and Hb concentration with a weak-moderate correlation coefficient (Table 6).

**Table 6:** Correlation between hemoglobin concentrations and basic sonographic parameters.

Bleeding group Correlation between Hb and sonographic parameters	Perimenopausal r (sig. 2-tailed)	Postmenopausal r (sig. 2-tailed)	Overall study r (sig. 2 tailed)
Uterine length	−0.19 (0.04)	−0.19 (0.15)	−**0.37 (<0.01)**^b^
Anterior-posterior diameter of uterine corpus	−0.17 (0.11)	−0.13 (0.33)	−**0.32 (<0.01)**^b^
Largest dimension of intra-cavitary uterine mass lesion	−**0.40 (0.03)**^a^	−0.11 (0.68)	−0.18 (0.24)
Endometrial thickness	0.04 (0.70)	−0.15 (0.28)	−0.02 (0.81)

Pearson correlations are shown.

a Correlation significant at the 0.05 level (two-tailed).

b Correlation significant at the 0.01 level (two-tailed).

## Discussion

### Paraclinical features in women with peri- and postmenopausal bleeding

#### Histopathological examination

As shown in Table 1, the most common cause of AUB in perimenopausal and postmenopausal women was EH, accounting for 60.7%, similar to findings reported by Ashour et al. (2017) and Bhansail et al. (2017).^18^^,^^19^ Other common pathologies in perimenopausal women with AUB were EP (15.2%) and EF (13.0%). After EH, EC was the second common pathology and was observed in approximately 38.3%, whereas EP and EF were observed in only 5%, of women with postmenopausal bleeding. Other causes of AUB, such as endometritis and endometrial hemorrhage, accounted for only 4%. A difference was observed among UIPs because of the difference in age. In postmenopausal women, because the endometrium undergoes a long period of exposure to risk factors, the etiology of PMB may be due to benign, such as atrophy, endometritis or EH, but these women show a higher percentage of malignancy than perimenopausal women.^19^, ^20^, ^21^, ^22^, ^23^ Because our study did not involve clinical intervention, only patients with histopathological results were recruited, and these results were compared with the ultrasound results. Participants lacking histopathological results were excluded from the study; therefore, endometrial atrophy and normal endometrium were rare in the present study. Women with postmenopausal bleeding had an EC rate of 38.3%, a value similar to those reported by Fatima et al. in Pakistan (30.55%) and Bohîlțea et al. in Romania (30.0%).^24^^,^^25^ However, the incidence of EC in our study was higher than those reported in other studies (17.5–18.0%).^26^^,^^27^ The different rates of EC might have been due to differences in population characteristics, periods and study samples. Moreover, because our hospitals are tertiary hospitals responsible for managing cancer diseases, the rate of EC treatment was higher than that in other hospitals in our region. In the malignant group, the most common type was adenocarcinoma, which accounted for more than 80% of cases.^27^^,^^28^ In our study, adenocarcinoma accounted for 88% of neoplasms. In the EH group, typical hyperplasia accounted for the majority (74.7%) of cases and had a frequency 3-fold higher than that of atypical hyperplasia. In a study by Bohitea et al. (2015), typical and atypical hyperplasia accounted for 46.7% and 8.2%, respectively.^25^

#### Hemoglobin concentration (Hb g/l) and anemia levels

**Figure 3:**
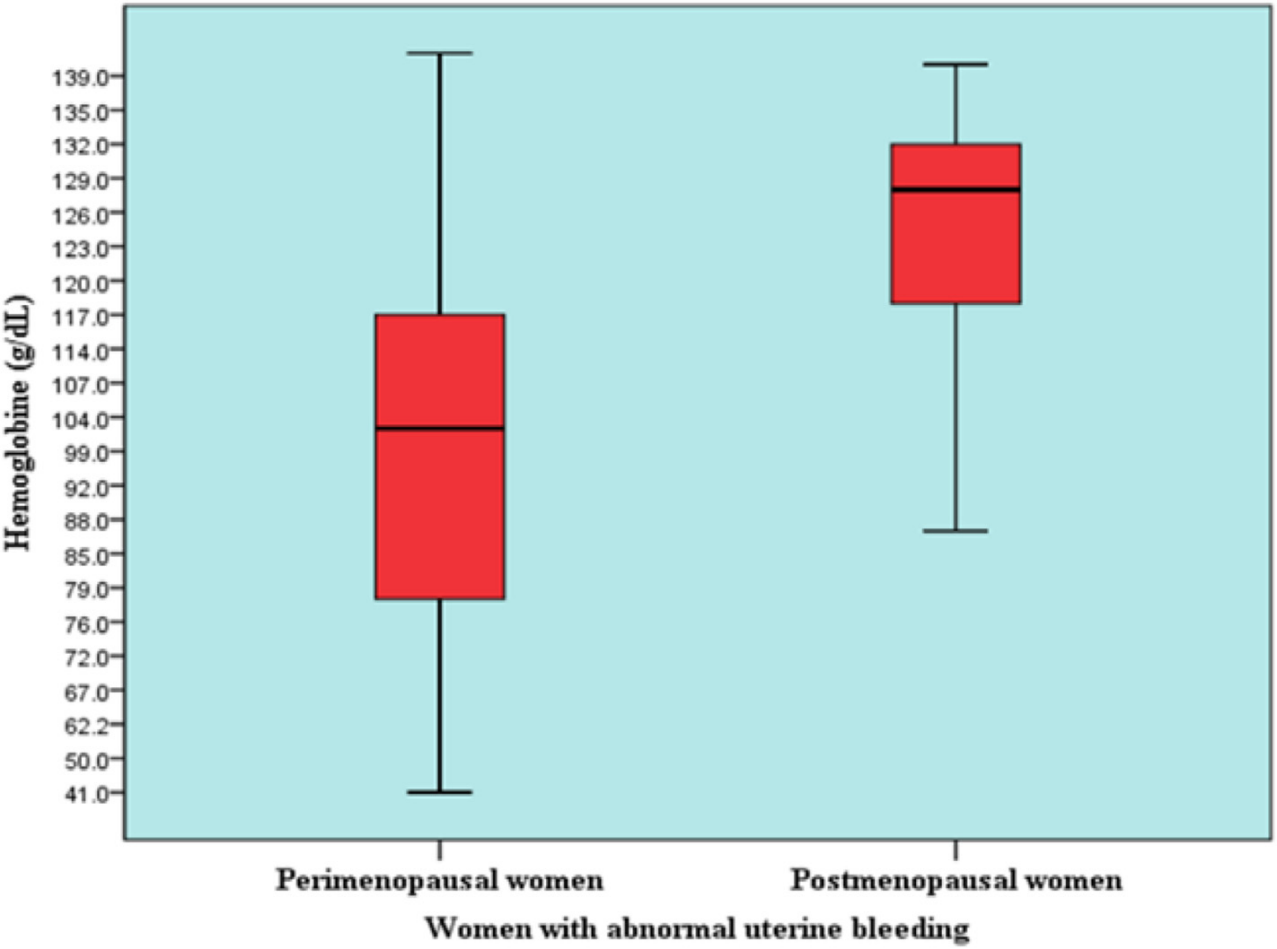
Mean concentration of Hb (g/l) in women with perimenopausal and postmenopausal bleeding.

According to anemia feature, the mean Hb concentration in our study was lower than that reported by Kucur et al. among 97 women with AUB: 109.07 ± 23.99 (g/l) compared with 117.5 ± 18.3 (g/l) (p < 0.0001).^29^ This difference might have been due to differences in race and diet. Moderate and severe anemia were common in perimenopausal women, accounting for 30.0% and 26.7%, respectively, in the anemia group (Table 2). A total of 20 cases (13.3%) of anemia required blood transfusion, with an average number of blood units of 3.45 ± 2.28, and a range of at least 2 units to as many as 10 units (250 ml/unit). In women with PMB, we observed no cases of severe anemia; 6/60 cases of moderate anemia accounted for 10.0%, mild anemia accounted for 16.7%, and no anemia classification (Hb > 120 g/l) was predominant (73.3%). Notably, the average concentration of Hb in the perimenopausal group was 99.41 g/l, compared with 123.55 g/l in the postmenopausal group (Figure 3). This finding might have been because perimenopausal women may be less concerned about bleeding during their menstrual period and may wait until symptoms are more severe before seeking medical attention, or because the amount of blood loss had not been properly assessed.^25^^,^^30^

### Basic ultrasonographic parameters in women with peri- and postmenopausal bleeding

#### Uterine size

The mean uterine size was smaller in postmenopausal than perimenopausal women, a finding completely compatible with anatomical and physiological characteristics. Although many mass-shaped lesions were observed, the AP diameter and uterine length in the group of perimenopausal women did not significantly differ between benign and malignant diseases (p > 0.05). In contrast, in women with postmenopausal bleeding, the uterine size was significantly larger in the malignant than the benign groups (p < 0.05). In a study by Ferrazi et al., the mean length of the uterus in the postmenopausal group was 65 (54–78) mm, a value smaller than the 72.54 ± 19.37 (40–140) mm in our study.^31^ Herein, the mean AP diameter in the postmenopausal bleeding group was 44.7 ± 11.8 (26–74) mm (Table 4).

#### Endometrial thickness

Endometrial thickness significantly differed between the benign and malignant group, particularly in postmenopausal women with vaginal bleeding (Table 4). This difference has also been reported in other studies.^12^^,^^32^

#### Uterine intracavitary lesion mass

Regardless of the mean size of the uterine intra-cavitary mass lesions, the largest lesions were EF (45.38 ± 25.68 mm), followed by EC (44.23 ± 20.94 mm) and EP (22.18 ± 11.67 mm). The smallest lesions were polypoid hyperplasia (13.14 ± 4.94 mm) (Table 5). The lesion in the malignant group with the mean largest size was 44.23 ± 20.94 mm, in 13 out of 25 cases of EC—more than the number of lesions in the benign group (two cases of polyps and one case of submucosal fibroids). This result differs from those of Yazdani et al. (2018), which indicated no difference in uterine size between the EC and benign group.^33^

In a study by Amreen et al. (2018), the mean polyp size was 15.29 mm.^34^ Cogendez et al. (2015) have reported a mean lesion size of 20.33 ± 10.26 mm and mean polyp size of 15.72 ± 7.39 mm. The mean subendometrial fibroid size was 30.56 ± 8.20 mm.^35^ Uterine fibroids are usually larger than polyps; therefore, Tamura-Sadamori et al. have concluded that if a lesion measures > 20 mm, it is significantly more likely to be a subendometrial fibroid than a polyp.^36^ Of sixteen cases of polyps, one case with a size of 4 mm was neglected and was later detected by hysteroscopy. The lesions in our study were larger than those in other studies: the largest submucosal fibroid measurement was 110 mm, possibly because a lack of access to health care services resulted in late diagnosis after the development of complications, or because, the patients allowed the tumors to develop for a long time period because of anxiety regarding surgery.

### Correlations between paraclinical features and basic ultrasonographic parameters

**Figure 4:**
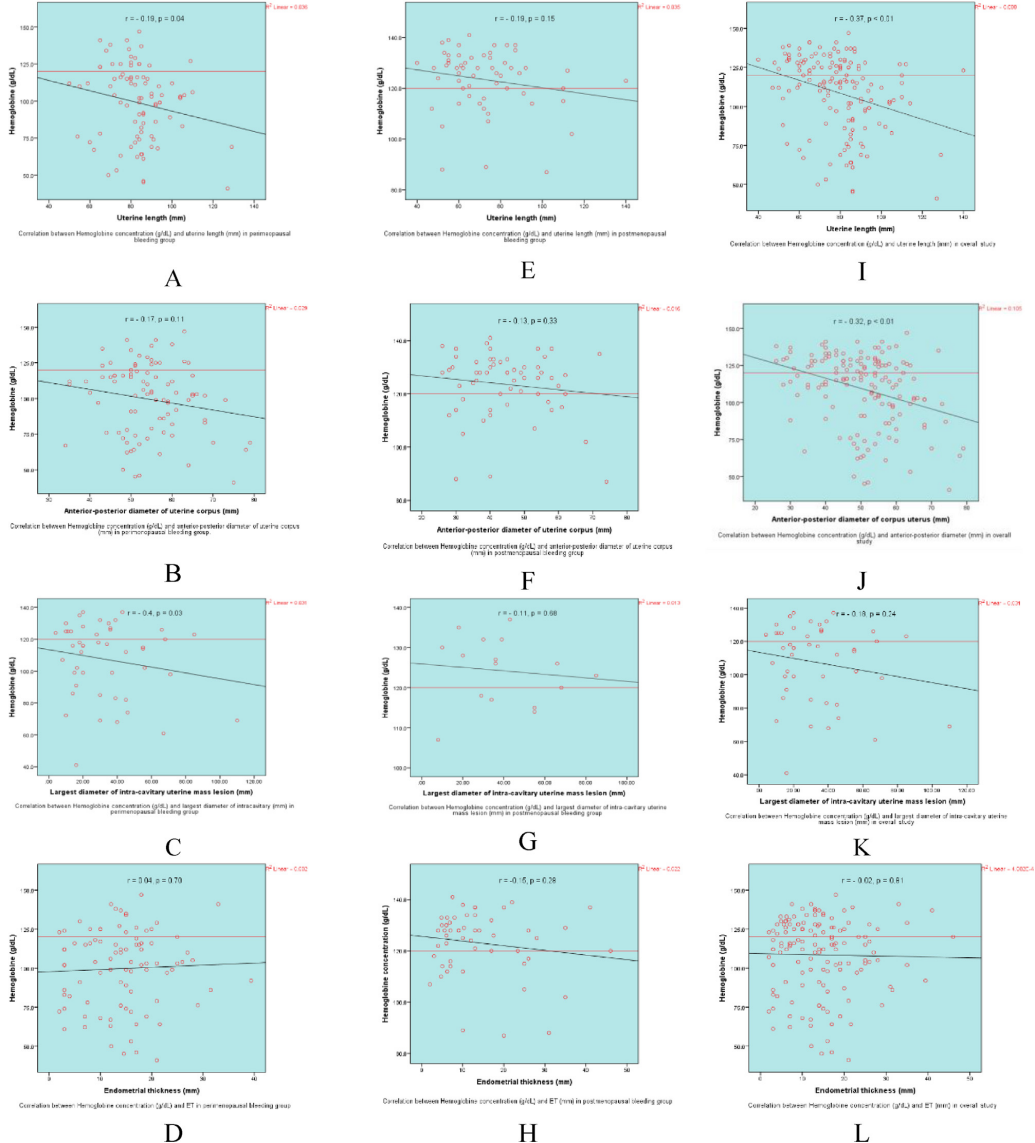
Correlation between hemoglobin concentration (g/l) and uterine length (mm), anterior-posterior diameter of the uterine corpus (mm), largest diameter of the uterine intracavitary mass lesion (mm) and endometrial thickness (mm) in the perimenopausal bleeding group (A–D), postmenopausal bleeding group (E–H) and overall study (I–L).

We found a weak-moderate negative correlation between Hb concentration (g/l) and the uterine length (mm) and AP diameter of the uterine corpus (mm) in the overall study. Notably, we observed a moderate negative correlation between Hb concentration and uterine intra-cavitary mass lesions in the perimenopausal bleeding group (P < 0.05). Interestingly, no significant correlation was observed between anemia and endometrial thickness (mm) (Table 6 and Figure 4). In 2006, Clancy et al. reported a positive correlation between ET and Hb, and concluded that a thicker endometrium is not associated with greater anemia. However, that study enrolled only 26 premenopausal women.^37^ In fact, some patients in our study had low endometrial thickness in cases with atrophy or endometritis, or had extrauterine lesions along with UIPs, and also had low Hb concentrations. These findings may be explained by ovulatory dysfunction leading to heavy menstrual bleeding or by endometrial thickness having been measured at the end of the menstrual period in perimenopausal women or after the absolutely sloughing of endometrial tissue following an uterine hemorrhage in postmenopausal bleeding women.^1^

Overall, endometrial hyperplasia should be carefully managed by clinicians to avoid progression to moderate-severe anemia in women with perimenopausal bleeding, and abnormal endometrium thickness is a risk factor for endometrial cancer in women with postmenopausal bleeding.^6^^,^^7^^,^^13^^,^^38^ Our study indicated no significant correlation between Hb concentration and endometrial thickness; however, the greater measured uterine size appeared to be associated with lower Hb levels. In fact, a large uterus may be accompanied by bulky fibroids, as well as other intrauterine masses, thus leading to severe hemorrhage and anemia.^39^ From a clinical perspective, a low Hb concentration might be predicted, and iron intake could be increased earlier to prevent anemia in these patients. However, more data are needed to confirm these findings.

### Strengths and limitations

This study described the rate of uterine intracavitary pathologies, and revealed the high prevalence of endometrial cancer as well as features of anemia in perimenopausal and women with postmenopausal bleeding in central Vietnam. Ultrasonography was performed by sonographers with 5 years’ experience at tertiary referral hospitals, and the results were analyzed on the basis of histopathological endpoints. Therefore, the analysis was accurate. Notably, our study found a correlation between Hb and several basic sonographic parameters, which had not previously been reported in the literature. Additionally, our findings demonstrated no significant correlation between Hb concentration and endometrial thickness. Nevertheless, this study included only patients who underwent ultrasonic scans and histopathological examination. Bias might have been present because of the of number of patients with vaginal bleeding without surgical intervention who were not included in this study. Histopathological endpoints did not include extensive analysis of subtypes of hyperplasia according to the classification of the World Health Organization in 1994 (WHO94), including simple hyperplasia without atypia, simple hyperplasia with atypia, complex hyperplasia without atypia, complex hyperplasia with atypia as well as concise subtypes of endometrial cancer.^40^ Additional limitations include the study's small sample size without a control group, and possible study bias due to different sonographic machines and sonographers. Further data are necessary to accurately evaluate these findings.

## Conclusion

Endometrial hyperplasia is the most common cause leading to moderate to severe anemia of intra-cavitary abnormalities. Uterine size, intrauterine mass and endometrial thickness were associated with malignant lesions in the postmenopausal bleeding group. A weak to moderately negative correlation was observed between Hb concentration and uterine size, but not endometrial thickness. Further studies are needed to validate these findings.

## Source of funding

This research did not receive any specific grant from funding agencies in the public, commercial or not for profit sectors.

## Conflict of interest

The authors have no conflict of interest to declare.

## Ethical approval

The study was approved by the institutional ethical committee under IRB approval number 1435/QD-DHYD.

## Consent to participate

Written informed consent was obtained from the patients for recruitment in the study.

## Consent for publication

Both authors consent to publication. This article was not published previously.

## Authors contributions

Conceptualization, P.N.N.; Data collection, P.N.N.; Investigation, P.N.N.; Methodology, P.N.N. and V.T.N.; Project administration, V.T.N. and P.N.N.; Resources, P.N.N.; Software, PN.N.; Supervision, V.T.N. and P.N.N.; Visualization, P.N.N. and V.T.N.; Writing-original draft, P.N.N.; Writing-review and editing, P.N.N. All authors have critically reviewed and approved the final draft and are responsible for the content and similarity index of the manuscript.

## Acknowledgment

We thank the patients who agreed to participate in our research and to the publication of their clinical data. We are also grateful to all teachers and colleagues working at Department of Gynecology, Department of Imaging Diagnostics and Department of Histopathology at Hue Central Hospital and Hue University Hospital, who provided us with images, cared for patients and shared their valuable experience in managing the clinical course.

## Availability of data and material

The datasets used and/or analyzed during the current study are available from the corresponding author on reasonable request.
